# A Subspace Based Transfer Joint Matching with Laplacian Regularization for Visual Domain Adaptation

**DOI:** 10.3390/s20164367

**Published:** 2020-08-05

**Authors:** Rakesh Kumar Sanodiya, Leehter Yao

**Affiliations:** Department of Electrical Engineering, National Taipei University of Technology, Taipei 10608, Taiwan; rakesh.pcs16@ntut.edu.tw

**Keywords:** domain adaptation, unsupervised discriminant analysis, transfer learning, classification, feature learning, instance re-weighting

## Abstract

In a real-world application, the images taken by different cameras with different conditions often incur illumination variation, low-resolution, different poses, blur, etc., which leads to a large distribution difference or gap between training (source) and test (target) images. This distribution gap is challenging for many primitive machine learning classification and clustering algorithms such as k-Nearest Neighbor (k-NN) and k-means. In order to minimize this distribution gap, we propose a novel Subspace based Transfer Joint Matching with Laplacian Regularization (STJML) method for visual domain adaptation by jointly matching the features and re-weighting the instances across different domains. Specifically, the proposed STJML-based method includes four key components: (1) considering subspaces of both domains; (2) instance re-weighting; (3) it simultaneously reduces the domain shift in both marginal distribution and conditional distribution between the source domain and the target domain; (4) preserving the original similarity of data points by using Laplacian regularization. Experiments on three popular real-world domain adaptation problem datasets demonstrate a significant performance improvement of our proposed method over published state-of-the-art primitive and domain adaptation methods.

## 1. Introduction

**Figure 1:**
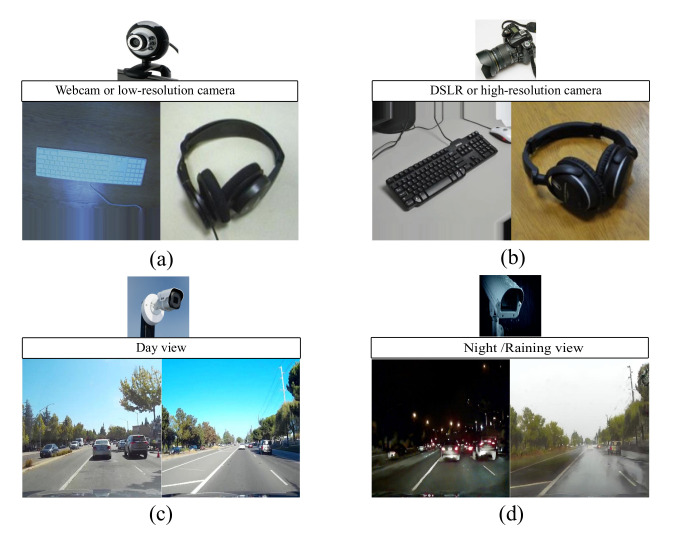
Shows different possibilities th at can cause distribution differences in the source domain (training) or target domain (test) images: (**a**) Images captured from low-resolution camera such as Webcam, (**b**) Images captured from high-resolution camera such as Digital Single-lens Reflex(DSLR), (**c**) Images captured in day time, and (**d**) Images captured in night or raining time.

Indoor–outdoor camera surveillance systems [1,2] are widely used in urban areas, railway stations, airports, smart homes, and supermarkets. These systems play an important role in security management and traffic management [3]. However, cameras with different properties and positions deployed in these systems can create a distribution difference among the capturing images. This difference leads to the system’s poor performance due to considering primitive machine learning algorithms for recognition [4]. For example, if a classifier (or primitive algorithm) is trained from source domain images (taken from a DSLR camera), then the trained classifier will not give as expected results when tested on the images collected from some other target domain (taken from webcam camera). A simple solution to improve the classifier’s performance is that it must only be trained with target domain images. However, there are no labeled images in the target domain in practice, and labeling the target domain images is a time-consuming process. Let us consider an example shown in Figure 1 to discuss in detail how the images collected from different environments can cause differences in distribution across domains. In Figure 1, various possibilities, which can cause distribution differences, are presented, such as (1) the images (keyboards and headphones) as shown in Figure 1a,b are collected from different quality cameras, i.e., low-quality camera (webcam camera) and high-quality camera (DSLR); (2) the images as shown in Figure 1c,d are taken with different weather conditions, i.e., a day or clear weather and night or rainy season.

Recently, the literature [2,4] has seen a growing interest in developing transfer learning (TL) or domain adaptation (DA) algorithms to minimize the distribution gap between domains, so that the structure or information available in the source domain can be effectively transferred to understand the structure available in the target domain. In previous work [5,6,7,8,9,10,11,12], two learning strategies for domain adaptation are considered independently: (1) instance re-weighting [9,10,11,12], which reduces the distribution gap between domains by re-weighting the source domain instances and then training the model with the re-weighted source domain data; (2) feature matching [5,6,8,13,14], which finds a common feature space across both domains by minimizing the distribution gap.

**Figure 2:**
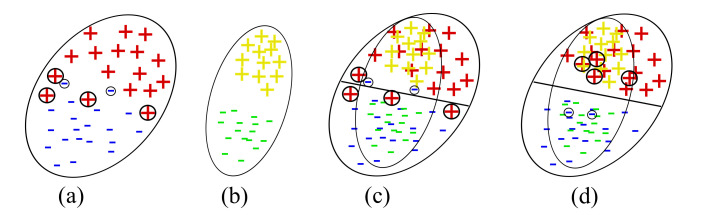
(**a**) Initial representation of source domain data with two classes (i.e., red plus (+) symbol and blue minus (−) symbol) in 2-d space, where the symbols with circles are outlier data samples. (**b**) Initial representation of target domain data with two classes (i.e., yellow plus (+) symbol and green minus (−) symbol) in 2-d space. (**c**) Data representation after application of only feature learning. (**d**) Data representation after application of jointly feature learning and instance re-weighting.

If the distribution difference between both domains is large enough, there will always be a situation where the source domain instances are not relevant to the target domain instance, even after finding a common feature location. In this situation, jointly optimizing instance re-weighting and feature matching is an important and unavoidable task for robust transfer learning. To understand a need for joint-learning instance re-weighting and feature matching more deeply, let us consider an example in which we have source domain data with outlier data samples (or irrelevant instances) as shown in Figure 2a and target domain data as shown in Figure 2b. In this case, if we lean only the common feature space between both domains by existing methods such as Joint Geometrical and Statistical Alignment (JGSA) [8] and Joint Distribution Adaptation (JDA) [6], the new representation of the source and target domain data is shown in Figure 2c, where it can be seen that the domain difference is still large for feature matching due to outlier data samples or irrelevant instances (the symbols with circles). However, if we jointly learn feature matching and instance re-weighting, the data representation is shown in Figure 2d, where it can be seen that all the outlier data samples are down-weighted to reduce domain difference further.

Fortunately, in the literature, there is a method called Transfer Joint Matching (TJM) that performs joint feature matching and instance re-weighting by down-weighting irrelevant features of the source domain [7]. However, only performing feature matching and instance re-weighting is insufficient to successfully transfer knowledge from the source domain to the target domain. Some other DA and TL methods consider other essential properties to minimize distribution differences between both domains. For example, the JDA method considers the conditional distribution in addition to the marginal distribution, and this distribution is needed if the data is conditionally or class-wise distributed. Subspace Alignment (SA) [15] makes use of subspaces (composed of ‘d’ eigenvectors induced by a Principle Component Analysis (PCA)), one for each domain and suggests minimizing the distribution difference between subspaces of both domains rather than the original space data. JGSA preserves source domain discriminant information, among other properties such as SA, marginal, and conditional distributions, to further improve the performance of JDA. However, the feature space obtained by JGSA is not notable because data samples in this space may lose their original similarity so that they can be easily misclassified by the classifier. Kernelized Unified Framework for Domain Adaptation (KUFDA) [16] improves JGSA by adopting the original similarity weight matrix term so that the sample does not lose its original similarity in the learned space. KUFDA follows most of the above discussed important properties but still suffers from outlier data samples, and this is due to not considering instance re-weighting term.

In this paper, to solve all of the above-discussed challenges and to efficiently transfer knowledge from the source domain to the target domain, we propose a novel Subspace based Transfer Joint Matching with Laplacian Regularization (STJML) method for visual domain adaptation by jointly matching the features and re-weighting instances across both the source and the target domains.

The major contributions of this work can be listed as follows:

- The proposed method STJML is the first framework that crosses the limits of all the comparative cutting edge methods, by considering all inevitable properties such as projecting both domain data into a low dimensional manifold, instance re-weighting, minimizing marginal and conditional distributions, and geometrical structure of both domains in a common framework.
- With the help of the t-SNE tool, to illustrate the reason for the inclusion of all the components (or inevitable properties), we have graphically visualized the features learned by the proposed method after excluding any component.

## 2. Related Work

Recently, various DA and TL approaches have been proposed for transferring structure or information from one domain to another domain in terms of features, instances, relational information, and parameters [4,9]. However, the TL approaches, which are closely related to our work, can be divided into three types: feature-based transfer learning [6], instance-based transfer learning [7], and metric-based transfer learning [9].

In the first type, the objective is to minimize the distribution difference between the source and the target domains based on feature learning. For example, Pan et al. [17] proposed a new dimensionality reduction method called maximum mean discrepancy embedding (MMDE) for minimizing the distribution gap between domains. MMDE learns a common feature space on the domains where the distance between distributions can be minimized while preserving data variance. Pan et al. [5] further extended the MMDE algorithm by proposing a new learning method called Transfer Component Analysis (TCA). TCA tries to learn a feature space across domains in a reproducing kernel Hilbert space using Maximum Mean Discrepancy (MMD). Therefore, with the new representation in this feature space, we can apply standard machine learning methods such as k-Nearest Neighbor (k-NN) and Support Vector Machine (SVM) to train classifiers in the source domain for use in the target domain. Long et al. [6] extends TCA by considering not only marginal distribution but also conditional distribution with the help of pseudo-labels in the target domain. Fernando et al. [18] introduced a subspace centric method called Subspace Alignment (SA). SA aims to align the source domain vectors (E) with the target domain one (F) with the help of a transformation matrix (M). Here, E and F can be obtained by Principle Component Analysis (PCA) on the source domain and the target domain, respectively. Shao et al. [19] proposed a low-rank transfer learning method to match both domain samples in the subspace for transferring knowledge. Zhang et al. [8] proposed a unified framework that minimizes the distribution gap between domains both statistically and geometrically, called Joint Geometrical and Statistical Alignment (JGSA). With the help of two coupled projections E (for source domain) and F (for target domain), JGSA projects the source domain and the target domain data into low dimensional feature space, where both domain samples are geometrically and statistically aligned.

In the second type, the objective is to re-weight the domain samples so as to minimize the distribution difference between both domain samples. The TrAdaBoost TL [10] method re-weights the source domain labeled data to filter samples that are most likely not from the target domain. In this way, the re-weighted source domain samples will create the same distribution found on the target domain. Finally, the re-weighted samples can be considered as additional training samples for learning the target domain classifier. As the primitive TrAdaBoost method is applicable to a classification problem, Pardoe et al. [11] extended this method by proposing ExpBoost.R2 and TrAdaBoost.R2 methods to deal with the regression problem.

In the final type, the target domain metric is to be learned by establishing a relationship between the source domain and the target domain tasks. Kulis et al. [20] introduced a method, called ARC-t, to learn a transformation matrix between the source domain and the target domain based on metric learning. Zhang et al. [21] proposed a transfer metric learning (TML) method by establishing the relationship between domains. Ding et al. [22] developed a robust transfer metric learning (RTML) method to effectively assist the unlabeled target learning by transferring the information from source domain labeled data.

## 3. A Subspace Based Transfer Joint Matching with Laplacian Regularization

This section presents the Subspace based Transfer Joint Matching with Laplacian Regularization (STJML) method in detail.

### 3.1. Problem Definition

To understand transfer learning or domain adaptation, the domain and the task must be explicitly defined. A domain D consists of two parts: features space X and a marginal probability distribution P(x), i.e., D={X,P(x)}, where x∈X. If there is a difference in their feature space or marginal distribution, the two domains are said to be different. Given a domain D, a task, that is denoted by T, also consists of two parts: a label space Y and a classifier function f(x), i.e., T={y,f(x)}, where y∈Y and classifier function f(x) predicts label of new instance *x*. This classifier function f(x) can also be interpreted as the conditional probability distribution, i.e., Q(y|x).

Transfer learning, given a labeled source domain $D_{s}=\left\{\left(x_{1},y_{1}\right),\cdots ,\left(x_{n_{s}},y_{n_{s}}\right)\right\}$ and unlabeled target domain $D_{t}=\left\{\left(x_{1}\right),\cdots ,\left(x_{n_{t}}\right)\right\}$ under the assumptions $X_{s}=X_{t},Y_{s}=Y_{t},P_{s}\left(x_{s}\right)\neq P_{t}\left(x_{t}\right),Q_{s}\left(y_{s}|x_{s}\right)\neq Q_{t}\left(y_{t}|x_{t}\right)$, aims to improve the performance of the target domain classifier function $f_{t}\left(x\right)$ in $D_{t}$ using the knowledge in $D_{s}$.

### 3.2. Formulation

To address the limitations of existing TL methods, the STJML method minimizes the distribution gap statistically and geometrically by working on the following components: finding both domain subspaces, matching features, instance re-weighting, and exploiting the similar geometrical property. In our proposed STJML approach, first, we exploit the subspaces of both domains and then with the help of common projection vector-matrix *Z* for both domains, perform feature matching, instance re-weighting, and the similar geometrical property exploitation in a Reproducing Kernel Hilbert Space (RKHS) to match both first and high-order statistics.

### 3.3. Subspace Generation

Even though both domain data lie in the same *D*-dimensional feature space, they are drawn according to different marginal distributions. Consequently, according to [15], instead of working on the original feature space, we need to work on more robust representations of both domain data to allow it to induce stronger classification, which is not subject to local perturbations. For this subspace generation, we use the Principle Component Analysis (PCA) technique, which selects ‘d’ eigenvectors corresponding to the ‘d’ largest eigenvalues. These ‘d’ eigenvectors are used to project original space data on it. For example, if a given input data matrix $X=\left[x_{1},x_{2},\cdots ,x_{n}\right]\in R^{D\times n}$ where $n=n_{s}+n_{t}$ and *D* is the dimension of each data sample in original space, then, the PCA generates the subspace matrix $X\in R^{d\times n}$ by projecting input data matrix (X) on selected ‘d’ eigenvectors.

### 3.4. Feature Transformation

As a dimensionality reduction method, such as PCA, can learn the transformed feature representation by reducing the reconstruction error of given data, it can also be utilized for the data reconstruction. Let us consider subspace data matrix $X\in R^{d\times n}$, data centering matrix $H=I-\frac{1}{n}1$, and 1 is a n×n matrix of ones. Thus, the covariance matrix of both domain subspace data matrix X can be calculated as $XHX^{T}$. The objective of PCA is to maximize both domain variances by finding an orthogonal transformation matrix $W\in R^{n\times \sigma }$, where σ is the selected number of eigenvectors on which subspace data matrix X to be projected. Thus,

(1) $$\begin{array}{c} \underset{W^{T}W=I}{\;\mathrm{Max}}\mathrm{tr}\left(W^{T}XHX^{T}W\right) \end{array}$$

where tr(.) is the trace of a matrix and *I* is an identity matrix. As the problem in Equation (1) is an eigendecomposition problem, it can easily be decomposed by eigendecomposition as $XHX^{T}W=W\Phi$, where $\Phi =\mathrm{diag}(\Phi_{1},\cdots ,\Phi_{\sigma })$ is the σ largest eigenval matrix. After projecting subspace data matrix X on the selected projection vectors matrix ($W_{\sigma }$) corresponding to top most σ largest eigenvalues matrix, the optimal σ-dimensional learned projection matrix $V=\left[v_{1},\cdots ,v_{\sigma }\right]=W_{\sigma }^{T}X$

To achieve our goal, we need to work in RKHS using some kernel function like linear, polynomial, Gaussian, etc. Let us consider the chosen kernel function θ, which maps the data sample *x* to θ(x), i.e., θ:x→θ(x), or $\theta \left(X\right)=[\theta \left(x_{1}\right),\cdots ,\theta \left(x_{n}\right)],$ and then the kernel matrix $K=\theta {\left(X\right)}^{T}\theta \left(X\right)\in R^{n\times n}$. After the application of the Representer theorem W=θ(X)Z, Equation (1) can be written as follows:

(2) $$\begin{array}{c} \underset{Z^{T}Z=I}{\;\mathrm{Max}}\mathrm{tr}\left(Z^{T}KHK^{T}Z\right) \end{array}$$

where $Z\in R^{n\times \sigma }$ is the transformation matrix, and the subspace embedding becomes $V=Z^{T}K$.

#### 3.4.1. Feature Matching with Marginal Distribution

However, even though maximizing the subspace data variance, the distribution difference between both domains will still be quite large. Therefore, the main problem is to minimize the distribution difference between them by applying an appropriate distance metric (or measure). There are many distance measures (such as the Kullback–Leibler (KL) divergence) that can be utilized to compute the appropriate distance between both domain samples. However, many of these methods are parameterized or require estimating the intermediate probability density [5]. Therefore, in this paper, we adopt a non-parametric distance estimate method called Maximum Mean Discrepancy (MMD) [23] to compare distribution difference in a Reproducing Kernel Hilbert Space (RKHS) [5]. MMD estimates the distance between the sample means of both domain data in the σ-dimensional embedding,

(3) $$\begin{array}{c} M_{d}={∥\frac{1}{n_{s}}\sum_{i=1}^{n_{s}}Z^{T}k_{i}-\frac{1}{n_{t}}\sum_{j=n_{s}+1}^{n}Z^{T}k_{j}∥}^{2}=\mathrm{tr}\left(Z^{T}KM^{d}K^{T}Z\right) \end{array}$$

where $M^{d}$ is the MMD matrix and can be determined as follows

(4) $$\begin{array}{c} M_{ij}^{d}=\left\{\begin{array}{cc} \frac{1}{n_{s}n_{s}} & k_{i},k_{j}\in D_{s}\\ \frac{1}{n_{t}n_{t}} & k_{i},k_{j}\in D_{t}\\ \frac{-1}{n_{s}n_{t}} & \mathrm{otherwise} \end{array}\right. \end{array}$$

#### 3.4.2. Feature Matching with Conditional Distribution

Minimizing the marginal distribution difference does not guarantee that the conditional distribution between both source and target domains will also be minimized. However, for robust transfer learning, minimizing the conditional distributions, i.e., $Q_{s}\left(y_{s}|x_{s}\right)$ and $Q_{t}\left(y_{t}|x_{t}\right)$ between both domains is required [24]. Reducing the conditional distribution is not a trivial process because there is no label data in the target domain. Therefore, we cannot model $Q_{t}\left(y_{t}|x_{t}\right)$ directly.

Long et al. [6] proposed a Joint Distribution Adaptation (JDA) method for modeling $Q_{t}\left(y_{t}|x_{t}\right)$ by generating pseudo labels of the target data. Initial pseudo labels for the target data can be generated by training the classifier with $X_{s}$ and $Y_{s}$ of the source domain, and testing the classifier on target domain subspace $X_{t}$. Now with $Y_{s}$, $X_{s}$, and pseudo labels, the conditional distribution between both domains can be minimized by modifying MMD to estimate distance between the class conditional distributions $Q_{s}\left(x_{s}|y_{s}=c\in \left\{1,\ldots ,C\right\}\right)$ and $Q_{t}\left(y_{t}|x_{t}=c\in \left\{1,\ldots ,C\right\}\right)$

(5) $$\begin{array}{c} C_{d}={∥\frac{1}{n_{s}^{c}}\sum_{k_{i}\in D_{s}^{c}}Z^{T}k_{i}-\frac{1}{n_{t}^{c}}\sum_{k_{j}\in D_{t}^{c}}Z^{T}k_{j}∥}^{2}=\mathrm{tr}\left(Z^{T}KM^{c}K^{T}Z\right) \end{array}$$

where $D_{s}^{c}=\left\{k_{i}:k_{i}\in D_{s}\wedge y\left(k_{i}\right)=c\right\}$ is the set of samples belongs to *c*th class in the source domain, $y(k_{i})$ is the true label of $k_{i}$, and $n_{s}^{c}=\left|D_{s}^{c}\right|$. Similarly, for the target domain, $D_{t}^{c}=\left\{k_{j}:k_{j}\in D_{t}\wedge \hat{y}\left(k_{j}\right)=c\right\}$ is the set of samples belongs to *c*th class in the target domain, $\hat{y}\left(k_{j}\right)$ is the pseudo label of $k_{j}$, and $n_{t}^{c}=\left|D_{t}^{c}\right|$. Thus, the MMD matrix $M^{c}$ with class labels of both domains can be determined as follows:

(6) $$\begin{array}{c} M_{ij}^{c}=\left\{\begin{array}{cc} \frac{1}{n_{s}^{c}n_{s}^{c}} & k_{i},k_{j}\in D_{s}^{c}\\ \frac{1}{n_{s}^{c}n_{t}^{c}} & k_{i},k_{j}\in D_{t}^{c}\\ \frac{-1}{n_{s}^{c}n_{t}^{c}} & \left\{\begin{array}{cc} k_{i}\in D_{s}^{c} & k_{j}\in D_{t}^{c}\\ k_{j}\in D_{s}^{c} & k_{i}\in D_{t}^{c} \end{array}\right.\\ 0 & \mathrm{otherwise} \end{array}\right. \end{array}$$

By minimizing Equation (5) such that Equation (2) is maximized, the conditional distributions between both the source and the target domains are drawn close with the new representation $V=Z^{T}K$. In each iteration, this representation *V* will be more robust till its convergence. As there is a difference in both the marginal and conditional distributions, the initial pseudo labels of the target domain are incorrect. However, we can still take advantage of them and improve the performance of target domain classifiers iteratively.

### 3.5. Instance Re-Weighting

However, matching features with marginal and conditional distributions is not sufficient for transfer learning, as it can only match first- and higher-order statistics. In particular, when the domain difference is significant enough, even in the feature learning, there will always be some source instances or samples that are not related to the target instance. In this condition, an instance re-weighting method with feature learning should also be included to deal with such a problem.

In this paper, we adopt a $L_{2,1}$-norm structured sparsity regularizer as proposed in [7]. This regularizer can introduce row-sparsity to the transformation matrix *Z*. Because each entry of the matrix *Z* corresponds to an example, row sparsity can substantially facilitate instance re-weighting. Thus, instance re-weighting regularizer can be defined as follows.

(7) $$\begin{array}{c} I_{r}={∥Z_{s}∥}_{2,1}+{∥Z_{t}∥}_{F}^{2} \end{array}$$

where $Z_{s}:=Z_{1:n_{s}}$ is the transformation matrix corresponding to the source samples, and $Z_{t}:=Z_{n_{s}+1:n_{s}+n_{t}}$ is the transformation matrix corresponding to the target samples. As the objective is to re-weight the source domain instances, we only impose $L_{2,1}$-norm on source domain. Thus, minimizing the Equation (7) such that Equation (2) is maximized, the source domain samples, which are similar (or dissimilar) to the target domain, are re-weighted with less (or greater) importance in the new learned space $V=Z^{T}K$.

### 3.6. Exploitation of Geometrical Structure with Laplacian Regularization

However, matching features and instance re-weighting are not enough to convey knowledge transfer by capturing the intrinsic structure of the source domain labeled samples and target domain unlabeled samples. In particular, labeled data samples of the source domain combined with unlabeled data samples of the target domain are used to construct a graph that sets the information of the neighborhood data samples. Here, the graph provides discrete approximations to the local geometry of the manifold data. With the help of the Laplacian regularization term L, the smooth penalty on the graph can be included. Basically, the term regularizer L allows us to incorporate prior knowledge on certain domains, i.e., nearby samples are likely to share same class labels [25].

Given a kernelized data matrix *K*, we can use a nn-nearest neighbor graph to establish a relationship between nearby data samples. Specifically, we draw an edge between any two samples i and j if $k_{i}$ and $k_{j}$ are “close”, i.e., $k_{i}$ and $k_{j}$ are among nn nearest neighbors of each other. Thus, the similarity weight matrix W can be determined as follows:

(8) $$\begin{array}{c} W_{ij}=\left\{\begin{array}{cc} 1 & \mathrm{if}\;k_{i}\in N_{nn}\left(k_{j}\right)\;\mathrm{or}\;k_{j}\in N_{nn}\left(k_{i}\right)\\ 0 & \mathrm{otherwise}\; \end{array}\right. \end{array}$$

where $N_{nn}\left(k_{j}\right)$ represents the set of nn nearest neighbors of $k_{i}$.

Here, two data samples are connected with an edge if they are likely to be from the same class. Thus, the regularizer term L can be defined as follows:

(9) $$\begin{array}{cc} L= & \sum_{ij}{\left(Z^{T}k_{i}-Z^{T}k_{j}\right)}^{2}W_{ij}\\ = & 2\sum_{i}Z^{T}k_{i}D_{ii}k^{T}Z-2\sum_{ij}Z^{T}k_{i}W_{ij}k_{j}^{T}Z\\ = & 2Z^{T}K\left(D-W\right)K^{T}Z\\ = & 2Z^{T}KLK^{T}Z \end{array}$$

where D is the diagonal matrix, i.e., $D_{ii}=\sum_{j}W_{ij}$ and *L* is the Laplacian matrix; L=D−W,

### 3.7. Overall Objective Function

The objective of this work is to minimize the distribution difference between domains by jointly matching the features of both domains and re-weighting the source domain samples, and preserving original similarity of both domain samples. So, by incorporating Equations (3), (5), (7), and (9), the proposed objective function can be obtained as follows:

(10) $$\begin{array}{c} \max_{Z^{T}KHK^{T}Z-1}\mathrm{tr}\left(Z^{T}K\left(\left(\delta -1\right)M_{d}+\delta C_{d}+\eta L\right)K^{T}Z\right)+\lambda \left({∥Z_{s}∥}_{2,1}+{∥Z_{t}∥}_{F}^{2}\right) \end{array}$$

where δ is a trade-off parameter, which balances the marginal and conditional distributions [13], η is the trade-off parameter that regularizes the Laplacian term, and λ is the regularization parameter to trade-off feature matching and instance re-weighting.

### 3.8. Optimization

By using the Lagrange multiplier Φ, Equation (10) can be written as follows:

(11) $$\begin{array}{c} L_{f}=\mathrm{tr}\left(Z^{T}K\left(\left(\delta -1\right)M^{d}+\delta C^{d}+\eta L\right)K^{T}Z\right)+\lambda \left({∥Z_{s}∥}_{2,1}+{∥Z_{t}∥}_{F}^{2}\right)+\mathrm{tr}\left(\left(I-Z^{T}KHK^{T}Z\right)\Phi \right) \end{array}$$

In order to find out an optimal value of the projection vector matrix *Z*, we partial derivative $L_{f}$ with respect to *Z* and equate it to zero as

(12) $$\begin{array}{c} \left(K\left(\left(\delta -1\right)M^{d}+\delta C^{d}+\eta L\right)K^{T}+\lambda G\right)Z=KHK^{T}Z\Phi \end{array}$$

${∥Z_{s}∥}_{2,1}$ is a non-smooth function at zero and its partial derivative can be computed as $\frac{\partial ({∥Z_{s}∥}_{2,1}+{∥Z_{t}∥}_{F}^{2})}{\partial Z}=2GZ$, where G is a diagonal subgradient matrix and its ith element can be calculated as

(13) $$\begin{array}{c} G_{ii}=\left\{\begin{array}{cc} \frac{1}{2∥a^{i}∥}, & k_{i}\in D_{s},a^{i}\neq 0\\ 0, & k_{i}\in D_{s},a^{i}=0\\ 1, & k_{i}\in D_{t} \end{array}\right. \end{array}$$

As the problem in Equation (12) is a generalized eigen decomposition problem, we can solve it and find $\Phi =diag(\phi_{1},\cdots ,\phi_{\sigma })$ (σ leading eigenvalues) and $Z=(z_{1},\cdots ,z_{\sigma })$ (σ leading eigenvectors). The pseudo code of our proposed method is given in Algorithm 1.

## 4. Experiments

In this section, we present a performance of the proposed STJML method by experimenting on various visual domain classification problems.

### 4.1. Data Preparation

We have considered three publicly image datasets: Office + Caltech10 with Speeded Up Robust Features (SURF), Office + Caltech10 with VGG-FC6 features, and Pose, Illumination, and Expression (PIE) face Recognition for experimentation. These data sets are well known in the domain adaptation methods and are widely considered in most recent works (such as [25,26]).

Caltech-256 has 30,607 images and 256 classes, while Office-31 is made of three object domains: DSLR (D), Amazon (A), and Webcam (W). It contains a total of 4652 images with 31 classes. As images in Office and Caltech-256 having different distributions, DA methods can help with cross-domain recognition. Since both the datasets contains 10 common classes, we considered Office + Caltech 10 datasets from [8], which has 12 tasks: A→D,⋯,C→W. For the purpose of experimentation, we considered both the SURF feature and Deep feature (VGG-FC6 features) of this dataset.

Carnegie Mellon University (CMU) PIE (Pose, Illumination, and Expression (PIE)) face dataset [27] contains over 40,000 facial images of 68 people. The images of each person were taken across 13 different poses, under 43 different illumination conditions, and with 4 different expressions. As there are many datasets of different poses, we considered only five poses such as C05, C07, C09, C27, and C29 for experimentation. Here each pose contains images with illumination variation and expression variation. Similar to Office + Caltech 10 datasets, 20 possible combinations of source and target domains or tasks such as C05→C07,⋯,C29→C27 can be constructed.

In this paper, we use notation P→Q to show knowledge transfer from source domain *P* to the target domain *Q*.

### 4.2. t-SNE Representation of Feature Spaces Learned by the Proposed Method (STJML)

**Figure 3:**
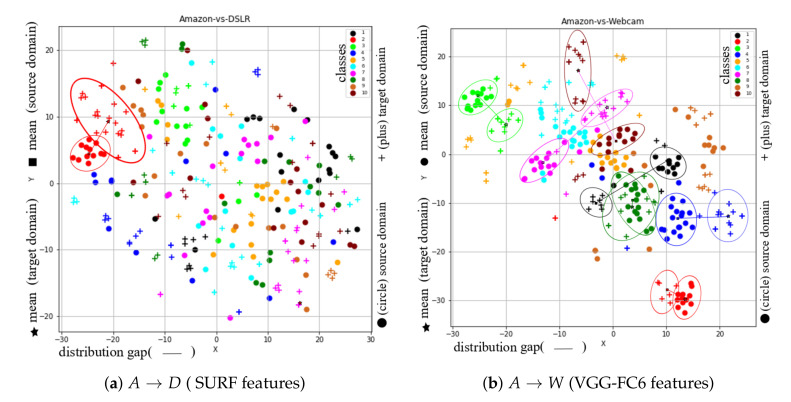
The t-SNE view of initial feature spaces on the tasks of Office + Caltech data set.

**Figure 4:**
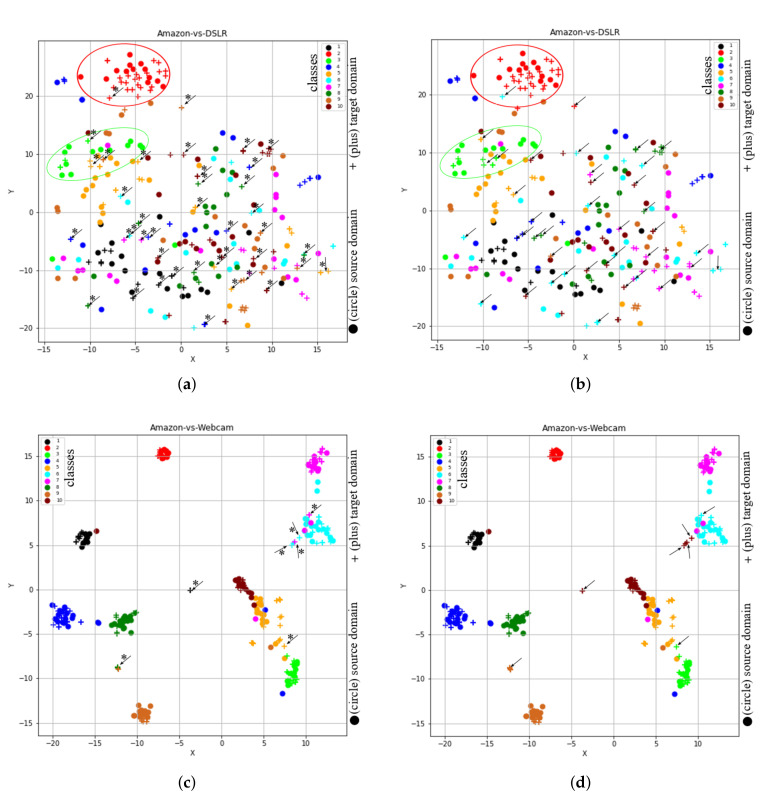
The t-SNE view of learned features from the proposed method Subspace based Transfer Joint Matching with Laplacian Regularization (STJML) after considering all the terms. (**a**) A→D (SURF features) with the predicted class labels for the target domain. (**b**) A→D (SURF features) with the given class labels for both domains. (**c**) A→W (VGG-FC6 features) with the predicted class labels for the target domain. (**d**) A→W (VGG-FC6 features) with the given class labels for both domains.

In order to visualize the learned feature space by our proposed method, we considered the t-SNE tool [28], through which high dimensional data is projected to 2-D space (or low dimensional space). To show t-SNE representation of feature spaces for tasks A→D (SURF features) and A→W (VGG-FC6 features), we randomly selected 150 samples from each domain and then used two different symbols (such as circles and pluses) to represent different domains and 10- different colors (‘black’, ‘red’, ‘lime’, ‘blue’, ‘orange’, ‘cyan’, ‘magenta’, ‘green’, ‘chocolate’, and ‘maroon’) to represent 10 different classes. Furthermore, to clearly understand the distribution differences between both domains, we used different colored ellipses to represent different classes’ variances belonging to different domains. We also used different colored lines to indicate the distribution gap between same class samples belong to different domains and different symbols such as square and star to represent the average point (or mean point) of each class in the source and target domains. For example, Figure 3a shows the initial feature representation of A→D task with SURF feature, where it can be seen that different class samples from different domains are too close together or there is no uniform cluster for different classes. Therefore, the classification or clustering algorithms can easily misclassify the samples that are too close or near the edge of their own clusters. However, due to the recent advancement in deep learning approaches, by which we can obtain deep features like VGG-FC6 features for the Office + Caltech10 dataset. The deep features (VGG-FC6) representation of task A→W for the Office + Caltech10 dataset is shown in Figure 3b. After comparing the representation of both types of features (as shown in Figure 3a), i.e., VGG-FC6 and SURF, it can be seen that the representation of VGG-FC6 features is much better than the SURF features (Figure 3a). Therefore, the performance of the primitive machine learning algorithm is better for deep learning features. The t-SNE representation of feature spaces learned by the proposed method (STJML) for both the tasks A→D (SURF features) and A→W (VGG-FC6 features) is shown in Figure 4.

To quantify misclassification samples in the learned feature space by the proposed method STJML for the task (A→D), we have shown two illustrations such as the first one with the predicted class labels for the target domain (as shown in Figure 4a) and the second one with the given class labels for both domains(as shown in Figure 4b). After carefully analyzing both the graphs, i.e., Figure 4a,b, many samples (highlighted by asterisks (*) and arrows (→))) are being misclassified by the proposed method STJML. However, If we compare the graph (as shown in Figure 3a) with the graph (as shown in Figure 4a), it can be seen that the distribution difference between the source domain samples and the target domain samples is minimized by a small margin. For example, in Figure 3a, it is visible that there is a distribution gap between the red class samples of the circle domain (or source domain) and the red class samples of the plus domain (or target domain). But, in Figure 4a, it can be seen that our proposed method STJML minimizes this distribution gap.

Similar to A→D (SURF features) task, if we compare graphs in Figure 3a and Figure 4c for A→W (VGG-FC6 features), it is observed that the distribution difference between both domains is satisfactorily reduced by the proposed method STJML. However, after comparing Figure 4c to Figure 4d, it can also be seen that there are only a few samples (marked by asterisks (*) and arrows (→))) which are being misclassified by our proposed method STJML.

### 4.3. What Happens if One Component Is Omitted from the Proposed Method (STJML)

To reveal the importance of including all of the above discussed components in our proposed method, we have experimented our proposed method on tasks A→D (SURF feature) and A→W (VGG-FC6 feature) by omitting any of its components at once. Therefore, by excluding any one component from our proposed method STJML, we can divide the proposed method into five new methods: ${\mathrm{STJML}}_{s}$(Omitting subspaces of both domains), ${\mathrm{STJML}}_{w}$ (Omitting instance-re-weighting term), ${\mathrm{STJML}}_{m}$ (Omitting marginal distribution term), ${\mathrm{STJML}}_{c}$ (Omitting conditional distribution term), and ${\mathrm{STJML}}_{l}$ (Omitting Laplacian regularization term).

#### 4.3.1. Omitting Consideration of Subspaces of Both Domains (${\mathrm{STJML}}_{s}$)

**Figure 5:**
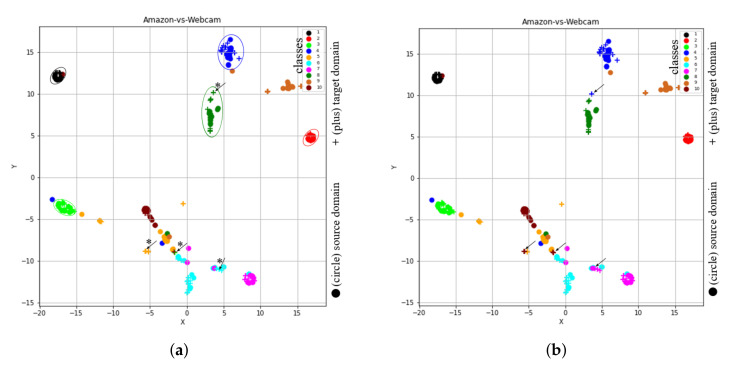
The t-SNE view of learned features from the proposed method after omitting the subspace of both domains. (**a**) A→W (VGG-FC6 features) with the predicted class labels for the target domain. (**b**) A→W (VGG-FC6 features) with the given class labels for both domains.

If we execute ${\mathrm{STJML}}_{s}$ on the original VGG-FC6 features of the task A→W, the learned feature representation is shown in Figure 5. In Figure 5, the first graph (i.e., Figure 5a) shows feature representation learned by method ${\mathrm{STJML}}_{s}$ with the given source domain labels and predicted target domain labels, while the second graph (i.e., Figure 5b) presents a representation of learned features with given both domain label information.

By comparing the representation of features for the task A→W (as shown in Figure 3b) with that of features learned by ${\mathrm{STJML}}_{s}$ (as shown in Figure 5a), the feature space learned by ${\mathrm{STJML}}_{s}$ is much better. From the comparison, it can also be seen that the distribution difference between both domains is minimum, as well as the distance between samples, belong to the same class is minimum, while the distance between different class samples is maximum. Thus, if this learned feature space is given to a classification algorithm, such as 1-NN classifier, the performance of the classifier (in terms of accuracy) will be 86.44%, which is accompanied by a 23% gain over the performance of the trained classifier with original feature space. Although the learned feature space is much better than the original feature space, there are some samples that are still being misclassified. In order to investigate those samples that are being misclassified by ${\mathrm{STJML}}_{s}$ method, we have also visualized these learned features (shown in Figure 5b) with the given both domain labels. If we compare the graphs as shown in Figure 5a,b, it can be observed that some sample class labels (as indicated with asterisks(*) and arrows(→)) predicted by the ${\mathrm{STJML}}_{s}$ are incorrect. Again if we compare the learned feature space (as shown in Figure 5a) by ${\mathrm{STJML}}_{s}$ method with the learned feature space (as shown in Figure 4c) by the proposed method STJML, the clusters for different classes obtained by our proposed method with all components are slightly better than those obtained by ${\mathrm{STJML}}_{s}$ method. For example, a black class cluster obtained by the STJML method is slightly distant from the maroon class samples, but that obtained by the ${\mathrm{STJML}}_{s}$ method collides with the samples with the maroon class. Similarly, the orange class cluster obtained by the ${\mathrm{STJML}}_{s}$ method is the worst compared to that obtained by the STJML method.

#### 4.3.2. Omitting Instance-Re-Weighting Term (${\mathrm{STJML}}_{w}$)

**Figure 6:**
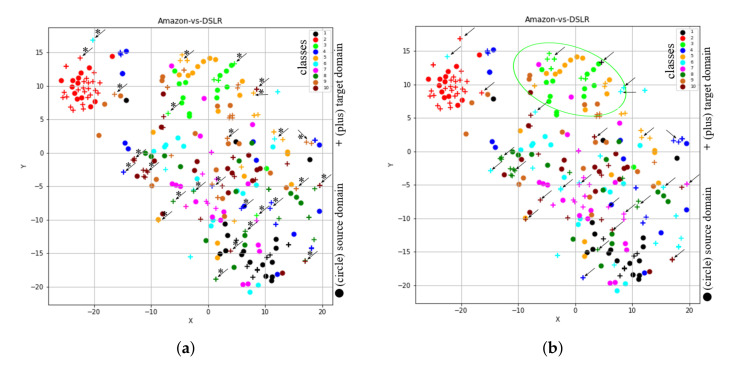
The t-SNE view of learned features from the proposed method after omitting the instance-re-weighting term. (**a**) A→D (SURF features) with the predicted class labels for the target domain. (**b**) A→D (SURF features) with the given class labels for both domains.

Since, the results obtained by both ${\mathrm{STJML}}_{w}$ and STJML methods for the task A→W (VGG-FC6 features) were similar, we considered another task A→D (SURF features) to show the effect of instance-re-weighting term. After executing ${\mathrm{STJML}}_{w}$ method on the task A→D (SURF features), the learned feature spaces are shown in Figure 6a,b. After comparing both the plots (as shown in Figure 6a,b), we can see that there are many samples (some of them we have highlighted by asterisks (*) and arrows (→)) which are being misclassified by the ${\mathrm{STJML}}_{w}$ method.

As the clusters obtained in Figure 6a,b by ${\mathrm{STJML}}_{w}$ method for the task A→D (SURF features) are not as good as obtained by the proposed method for the task A→W (VGG-FC6 features), we compare the graph (as shown in Figure 6b) obtained by ${\mathrm{STJML}}_{w}$ method with the graph (as shown in Figure 4b) obtained by the STJML method. After comparing both the plots in Figure 4b and Figure 6b, it can be concluded that some of the samples of source domain for ’lime’ colored class in Figure 6b (look at ‘lime’ colored ellipse) are not efficiently down weighted as compared to the graph in Figure 4b. Therefore, the performance of ${\mathrm{STJML}}_{w}$ method (which is 41.70% accuracy) is not as good as the STJML method (which is 49.10%).

#### 4.3.3. Omitting Marginal Distribution Term (${\mathrm{STJML}}_{m}$)

**Figure 7:**
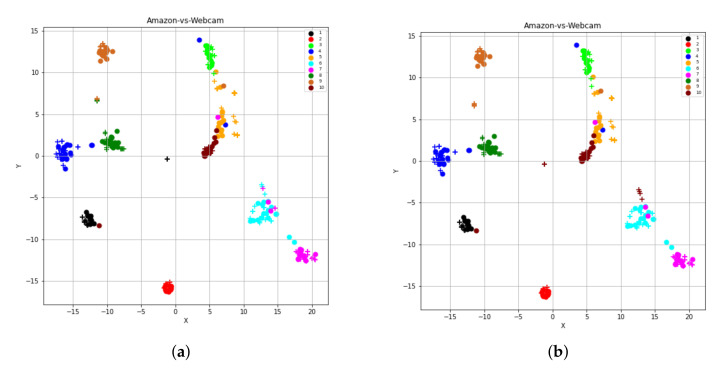
The t-SNE view of learned features from the proposed method after omitting the marginal distribution term. (**a**) A→W (VGG-FC6 features) with the predicted class labels for the target domain. (**b**) A→W (VGG-FC6 features) with the given class labels for both domains.

If we omit consideration of the marginal distribution from our proposed method STJML, the t-SNE views of learned feature spaces by ${\mathrm{STJML}}_{m}$ for A→W (VGG-FC6 features) task are shown in Figure 7a,b. After excluding the marginal distribution, the ${\mathrm{STJML}}_{m}$ method achieves 90.85% accuracy, which is similar to the accuracy achieved by the STJML method. Thus, it can be concluded here that even after dropping this distribution, the STJML method does not have much effect on its performance. Moreover, after carefully looking the graphs learned by ${\mathrm{STJML}}_{m}$ (as shown in Figure 7a,b) and STJML (as shown in Figure 4c,d) methods, we find that the graphs learned by both the methods are almost similar.

#### 4.3.4. Omitting Conditional Distribution Term (${\mathrm{STJML}}_{c}$)

**Figure 8:**
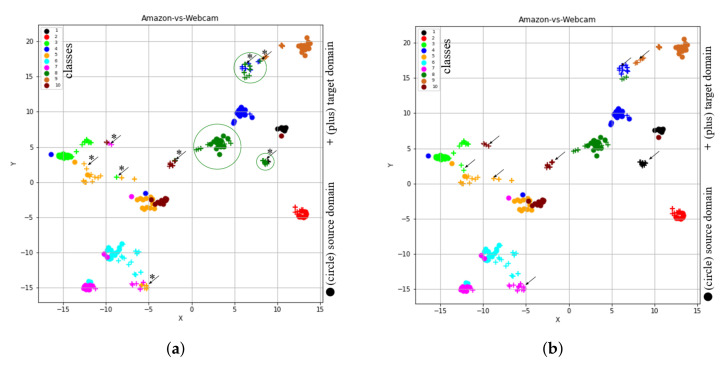
The t-SNE view of learned features from the proposed method after omitting the conditional distribution term. (**a**) A→W (VGG-FC6 features) with the predicted class labels for the target domain. (**b**) A→W (VGG-FC6 features) with the given class labels for both domains.

If we exclude consideration of the conditional distribution from our proposed method STJML, the t-SNE views of learned feature spaces by ${\mathrm{STJML}}_{c}$ for A→W (VGG-FC6 features) task are shown in Figure 8a,b. Without including this conditional distribution term in our proposed method STJML, the ${\mathrm{STJML}}_{c}$ approach achieves 73.90% accuracy, which is much lower than the accuracy (90.85%) achieved by the STJML method. Therefore, we can say that this term greatly impacts the performance of the proposed STJML method if it is not included. If we compare the graph (as shown in Figure 4c) obtained by the STJML method to the graph (as shown in Figure 8a) obtained by ${\mathrm{STJML}}_{c}$, it can be seen that the distribution difference between both domains has not been effectively reduced by the ${\mathrm{STJML}}_{c}$ method. For example, the distribution difference between green colored class samples of both domains (plus and circle) is not minimized in Figure 8a (i.e., all the green colored class samples are distributed in different green colored circles), but it can be seen in Figure 4c that all green colored class samples are with in a cluster. Because of not minimizing the distribution gap by the ${\mathrm{STJML}}_{c}$ method, we can see that some samples that are being misclassified in Figure 8a (as highlighted by asterisks (*) and arrows (→)) after comparing with the graph in Figure 8b.

#### 4.3.5. Omitting Laplacian Regularization Term (${\mathrm{STJML}}_{l}$)

**Figure 9:**
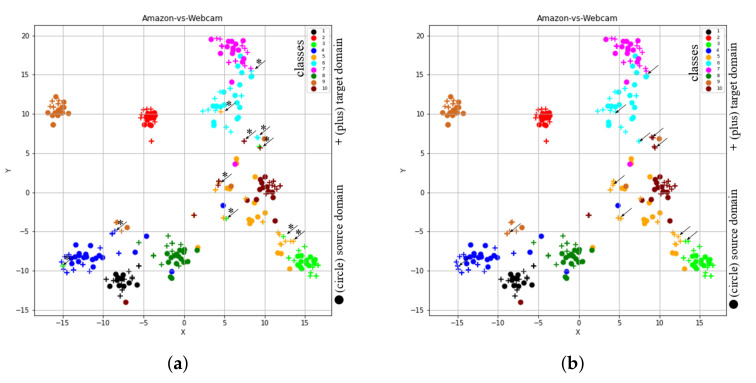
The t-SNE view of learned features from the proposed method after omitting the Laplacian distribution term. (**a**) A→W (VGG-FC6 features) with the predicted class labels for the target domain. (**b**) A→W (VGG-FC6 features) with the given class labels for both domain.

The samples that are supposed to loss their original similarity in the leaned feature space can preserve their original similarity by adding the Laplacian regularization term. As a result, samples that were supposed to go far away from their respective groups or clusters may come closer together. Thus, in order to see the impact of this term, we omit this term from the proposed method STJML and execute the algorithm. The t-SNE representation of learned feature spaces by ${\mathrm{STJML}}_{l}$ is depicted in Figure 9a,b. If we compare the graphs generated by ${\mathrm{STJML}}_{l}$ (as depicted in Figure 9a,b) with the graphs generated by STJML (as depicted in Figure 4c,d), it can be seen that the samples in each class cluster generated by ${\mathrm{STJML}}_{l}$ are widely spread around their mean point, but they are less spread in the cluster generated by STJML. Therefore, the performance (82.03% accuracy) of ${\mathrm{STJML}}_{l}$ method is slightly lower than the STJML method. After comparing the graphs in Figure 9a,b, we can see that some samples (highlighted by asterisks (*) and arrows (→))) are being misclassified by ${\mathrm{STJML}}_{l}$ method.

### 4.4. Comparison with State-Of-The-Art Methods

The proposed STJML method was verified and compared with many state-of-the-art primitive and domain adaptation algorithms. A brief description of all the comparative methods is as follows:

- NN, PCA+1NN, and SVM: These are the traditional machine learning algorithms, which assume that both training and test data should follow a uniform distribution.
- Transfer Component Analysis (TCA) [5]: TCA is a feature transformation technique that aligns only the marginal distribution of both domains.
- Joint Distribution Alignment (JDA) [6]: This method aligns both the marginal and conditional distributions of both domains.
- Geodesic Flow Kernel (GFK) [29]: In order to characterize the changes of geometric and statistical properties from the source domain to the target domain, GFK integrates countless subspaces.
- Transfer Joint Matching (TJM) [7]: This method demonstrates both feature learning and instance re-weighting for minimizing distribution differences between both domains.
- Subspace Alignment (SA) [15]: SA first projects both domain samples into lower-dimensional subspaces, then aligns both domains.
- Scatter Component Analysis (SCA) [30]: This method is based on a simple geometrical measure, i.e., scatter.
- CORAL [31]: It aligns both domain covariance matrices.
- Adaptation Regularization (ARTL) [14]: This method learns domain classifier in original space.
- Cross-Domain Metric Learning (CDML) [32]: It is a novel metric learning algorithm to transfer knowledge in an information-theoretic setting.
- Close yet Discriminative DA(CDDA), Geometry Aware DA (GA-DA), and Discriminative and Geometry Aware DA (DGA-DA) [33]: CDDA enhances the performance of the JDA method by incorporating a new repulsive force objective into its model to improve the discriminative power of the common feature subspace. Likewise, the GA-DA method includes the original parity of a data point in CDDA to improve its performance. Then, finally, the GA-DA method involves preserving the discriminative information term to improve the DGA-DA method performance further.
- Invariant Latent Space (ILS) [34]: This TL method makes use of the Riemannian optimization methods to match statistical properties.
- Balanced Distribution Adaptation (BDA) [35]: It is a novel TL approach to adaptively balance both the marginal and conditional distributions of both domain data.
- Joint Geometrical and Statistical Alignment (JGSA) [8]: It extends the JDA by adopting two projection vector matrices and considering subspace alignment and source discriminant information.
- Robust Transfer Metric Learning (RTML) [22]: This method considers two directions, i.e., sample space and feature space, to mitigate the distribution gap.
- Domain Invariant and Class Discriminative (DICD) [36]: This DA method is to learn a latent feature space while preserving important data properties.
- Explicit Map-based Feature Selection (EMFS) [37]: It attempts to: (1) reveal high-order invariant features by explicit feature map, (2) integrate feature learning and model learning, and (3) remove non-discriminative features from invariant features.
- Domain Irrelevant Class clustering (DICE) [38]: This method specifically deals with the intra-domain structure for the target domain in addition to other common properties.
- Linear Discriminant Analysis-inspired Domain Adaptation (LDADA) [39]: The key insight of this approach is to leverage the discriminative information from the target task, even when the target domain labels are not given.
- Kernelized Unified Framework for Domain Adaptation (KUFDA) [16]: This TL method improves the JGSA method by adding the Laplacian regularization term.

### 4.5. Parameter Sensitivity

Our proposed STJML method contains various parameters such as nn,k,σ,η,λ, and δ, along with other state-of-the-art domain adaptation methods [5,8,13,16]. Similar to previous methods [14,40], we also need to analyze the parameter sensitivity of the STJML method on all possible tasks of both datasets to validate that an appropriate value of each parameter can be chosen to obtain satisfactory performance. Analyzing the parameter sensitivity of STJML, we vary one parameter value and keep the other parameter values constant. For example, we vary parameter value *k* from 1 to 10 with an interval of 1 and keep other parameter values $nn=1,\sigma =100,\eta =10^{-1},\lambda =10^{-3},$ and δ=0.5 constant. Here, we have provided a description of each parameter and performed a parameter sensitivity test for all the considered datasets. But, we have shown parameter sensitivity analysis graphs for Office + Caltech10 with VGG-FC6 features and PIE face datasets. The description and possible values of each parameter are as follows:

#### 4.5.1. *k* Parameter

**Figure 10:**
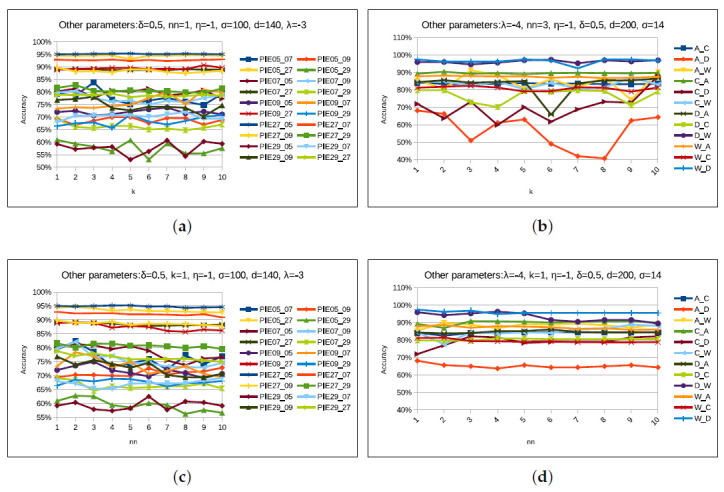
Performance of the proposed STJML method, varying the values of the parameters ‘*k*’ and ‘nn’ on all tasks in both the datasets. (**a**) *k*-Pose, Illumination, and Expression (PIE) face dataset. (**b**) *k*-Office + Caltech10 with VGG-FC6 features dataset. (**c**) nn-PIE face dataset. (**d**) nn-Office + Caltech10 with VGG-FC6 features dataset.

In our proposed method, we considered the k-NN classifier to predict the label of the target domain, and the performance of this classifier depends on an appropriate value of parameter *k* for each task. Therefore, we need to find out its proper value for each task of the datasets. For each task of the considered datasets, we varied *k* value from 1 to 10 with an interval of 1 and keep other parameter values constant as shown in Figure 10a,b. The resultant graphs for Office + Caltech10 with VGG-FC6 features and PIE face datasets are shown in Figure 10a,b. From Figure 10a,b, it can be seen that the STJML method outperforms at k=1 for most of the tasks of both the datasets. Therefore, we keep k=1 for most of the tasks of all the datasets except some tasks such as 9→7, 9→27, 9→29, 27→7, 27→09, C→D(VGG−FC6featurestask), C→A(SURFfeaturestask), C→W(SURFfeaturestask), D→A(SURFfeaturestask), D→C(SURFfeaturestask), W→A (SURF features task), but for these tasks k-parameter values are kept 9, 9, 5, 10, 10, 9, 3, 3, 10, 3, and 2, respectively.

#### 4.5.2. nn Parameter

Similar to the parameter *k*, we require an appropriate value of parameter nn for construing the Laplacian graph as discussed in Section 3.6. So, we vary nn value from 1 to 10 and keep other parameter values constant. Here also, in Figure 10c,d, there is no unique value of nn for which STJML is outperforming for all tasks of Office + Caltech with the SURF features dataset. However, STJML is outperforming for the values (1 and 2) of nn for the tasks of PIE dataset. Therefore, we keep nn=1 for all tasks (except 5→27(nn=2),7→5(nn=2), and 7→09(nn=2)) of PIE face dataset. Similarly, we keep nn=3 for all tasks (except A→C(nn=1) and A→W(nn=7)) of Office + Caltech with VGG-FC6 features dataset, but nn=10 for all tasks (except A→D(nn=1),A→W(nn=1),C→A(nn=8),C→D(nn=2), and W→C(nn=1)) of Office + Caltech with SURF features dataset.

#### 4.5.3. δ Parameter

**Figure 11:**
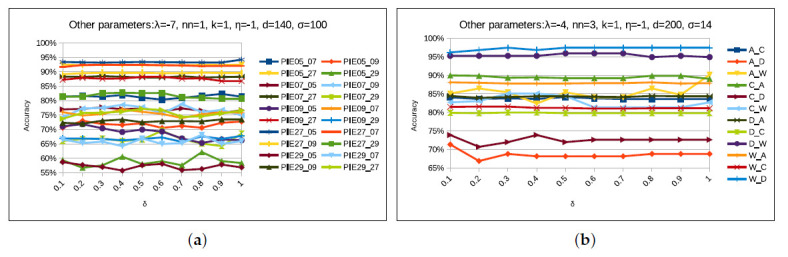
Performance of the proposed STJML method, varying the values of the parameter δ on all tasks in both the datasets. (**a**) δ-PIE face dataset. (**b**) δ-Office + Caltech10 with VGG-FC6 features dataset.

This parameter quantitatively evaluates the importance of aligning marginal and conditional distributions in domain adaptation. In this evaluation, the existing work [6,8] in DA fails by the assumption that both distributions are equally important. However, this assumption may not be true for real-world problems. Wang et al. [13] introduced the adaptive factor parameter to measure the importance of these two distributions dynamically. However, in this paper, we manually performed the parameter sensitivity tests to ascertain a reasonable value of this factor for each task. Thus, we varied its value from 0 to 1 with an interval of 0.1, and the resulted graphs are shown in Figure 11. It is clearly shown in Figure 11, that STJML performs well for different values of δ for different tasks. Thus, to achieve best performance of the STJML method, we keep δ=0.5 for all tasks (except 5→7(δ=0.9) and 7→29(δ=0.6)) of PIE face dataset. Similarly, we keep δ=0.5 for all tasks (except A→W(δ=0.9)) of Office + Caltech with VGG-FC6 dataset, but δ=0.9 for all tasks (except C→W(δ=0.9)) of Office + Caltech with SURF features dataset.

#### 4.5.4. Parameter: λ and η

**Figure 12:**
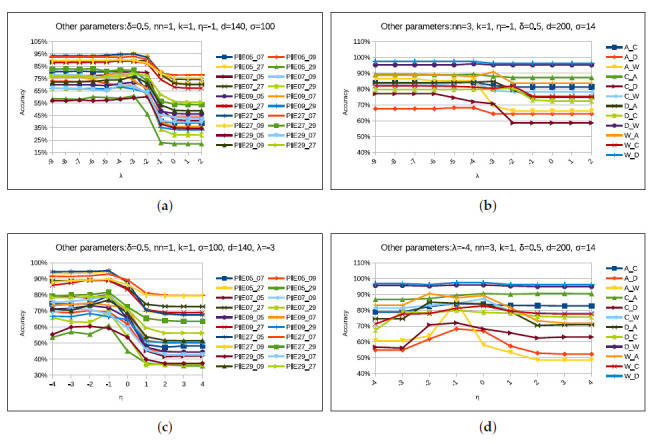
Performance of the proposed STJML method, varying the values of the parameters λ and η on all tasks in both the datasets. (**a**) λ-PIE face dataset. (**b**) λ-Office + Caltech10 with VGG-FC6 features dataset. (**c**) η-PIE face dataset. (**d**) η-Office + Caltech10 with VGG-FC6 features dataset.

As parameter λ is the trade-off parameter which regularizes the instance re-weighting term, we vary its values from $10^{-9}$ to $10^{2}$ and keep other parameter values constant as shown in Figure 12a,b, where the x-axis represents numbers from ‘−n’ to ‘n’, which are actually numbers from $10^{-n}$ to $10^{n}$. In Figure 12a,b, it is shown that STJML performs well for $10^{-4}$ and $10^{-3}$ for most of the tasks of both datasets. For better accuracy with respect to this parameter, we keep $\lambda =10^{-3}$ for all tasks (except $5\to 7\left(\lambda =10^{-7}\right),5\to 9\left(\lambda =10^{-6}\right),7\to 29\left(\lambda =10^{-7}\right),$ and $27\to 29(\lambda =10^{-6}))$ of PIE face dataset. But, we keep $\lambda =10^{-4}$ for all tasks (except $A\to W(\lambda =10^{-1}))$ of Office + Caltech with VGG-FC6 features dataset and $\lambda =10^{-1}$ for all tasks (except $A\to D\left(\lambda =10^{-3}\right),A\to W\left(\lambda =10^{-2}\right),D\to A\left(\lambda =10^{-4}\right),D\to C\left(\lambda =10^{-3}\right),W\to C\left(\lambda =10^{-3}\right)$, and $W\to D(\lambda =10^{-3}))$ of Office + Caltech with SURF features dataset.

Similar to the parameter λ, we also vary η parameter values from $10^{-4}$ to $10^{-4}$ and keep other parameter values constant as shown in Figure 12c,d. From Figure 12c,d, it can be determined that STJML outperforms at $10^{-1}$ for all tasks of PIE face dataset, while at $10^{-1}$ for some tasks of Office + Caltech dataset with VGG-FC6 features. For better performance, we keep $\eta =10^{-1}$ for all tasks (except $C\to A(\eta =10^{0})$ and $W\to A(\eta =10^{-2})$ tasks of VGG-FC6 features, and $D\to W(\eta =10^{0})$ and $W\to C(\eta =10^{0})$ tasks of SURF features) of considered datasets.

#### 4.5.5. Parameter: σ

**Figure 13:**
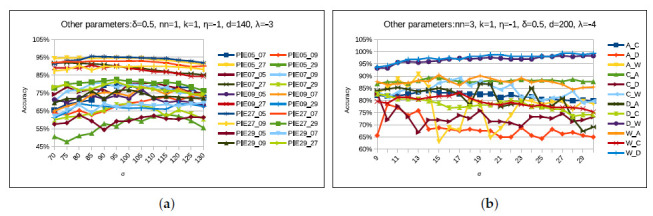
Performance of the proposed STJML method, varying the values of the parameter σ on all tasks in both the datasets. (**a**) σ-PIE face dataset. (**b**) σ-Office + Caltech10 with VGG-FC6 features dataset.

As the performance of the proposed STJML method depends on choosing the eigenvectors (σ) corresponding to the leading eigenvalues, we ran STJML with varying values of σ (70 to 130 with an interval of 5 for PIE dataset and 9 to 30 with an interval of 3 for Office + Caltech dataset ) and report the results in Figure 13a,b. We plot classification accuracy graph with respect to different values of σ in Figure 13a,b. In Figure 13a,b, it can be seen that proposed STJML the gives best accuracy for different values of this parameter for different tasks of both the datasets. For better accuracy with respect to this parameter, we keep σ=100 for all tasks (except 5→29(σ=115), 7→27(σ=75), 9→05(σ=105), 27→05(σ=85), and 27→09(σ=80)) of PIE face dataset. We keep σ=30 for all tasks of Office + Caltech with SURF features dataset, and σ=14 for all tasks (except A→C(σ=27), A→D(σ=20), A→W(σ=20), D→W(σ=30), D→C(σ=13), and W→A(σ=13) of Office + Caltech with VGG-FC6 features dataset.

#### 4.5.6. Parameter: *d*

In order to find a low dimensional subspace for both the domains, we need to project original data from *D*-dimensional space to a *d*-dimensional subspace. However, projecting data from *D*-dimensional space to a *d*-dimensional subspace, it can lose some information. Therefore, we need to find the appropriate value of *d*, so that the original information of both domains can remain in the low dimensional space as well.

Like other parameters, we also vary its value and keep other parameter values constant, and find that the proposed STJML method outperforms at d=140 for all tasks of the PIE face dataset, while d=100 for SURF features and d=200 for VGG-FC6 features of the Office + Caltech datasets.

### 4.6. Experimental Setup

To show the strength of the STJML method over previous state-of-the-art methods, we considered 12 tasks of Office + Caltech10 with SURF feature, 12 tasks of Office + Caltech10 with VGG-FC6 features, and 20 tasks of PIE face datasets. With the help of the parameter sensitivity test, we explored an appropriate value of each parameter of the STJML method and then used those values to ascertain the proposed method’s accuracy for each task of the considered dataset. Thus, after experimenting on the proposed STJML method, the accuracy of each task of all datasets is stated in Table 1, Table 2 and Table 3. However, for the accuracy of other comparative methods in Table 1, Table 2 and Table 3, we have derived directly from their respective papers or previous papers [5,7,8,13,14,16,37,38].

### 4.7. Experimental Results and Analysis

The recognition performance of the proposed method and the other compared state-of-the-art methods on three widely used domain adaptation datasets is reported in Table 1, Table 2 and Table 3. From the results reported in Table 1, Table 2 and Table 3, we can conclude the following observations:

- Primitive machine learning approaches such as NN, PCA, and SVM are not performing well due to the distribution gap between training (source data) and testing (target data) datasets.
- Among domain adaptation methods, the GFK method’s performance is worse for an average accuracy of all tasks in the Office + Caltech dataset for both SURF and VGG-FC6 features.
- The JDA method’s performance for all the three datasets is higher than that of the TCA method because it adopts the conditional distribution in addition to the marginal distribution.
- The ILS method works well compared to other subalignment methods (such as SA, GFK, and CORAL) because of considering the more robust discriminative loss function for the Office + Caltech dataset with deeper features.
- As TJM adopts the term instance re-weighting, its performance is better than other DA methods such as TCA, GFK, JDA, SA, CORAL, ILS, and BDA for the deep features of Office + Caltech dataset. However, for the SURF features, TJM gives better average accuracy than SCA, ARTL, GFK, and TCA, but performs poorly compared to JGSA, CORAL, LDADA, DICE, RTML, ILS, and JDA.
- The average accuracy (65.09%) of the DGA-DA method for all tasks in the PIE face dataset is higher than that of other methods (i.e., TCA, JDA, CDML, TJM, TDA-AL, CDDA, BDA, RTML, EMFS, and LDADA) because it involves novel repulsive force term and the Laplacian regularization term.
- Since JGSA improves JDA by considering two projection vector matrices and preserving source domain discriminant information, the mean accuracy (82.60%) of the JGSA method for all tasks in the Office + Caltech dataset with deep features is higher than other methods (i.e., TCA, JDA, GFK, SA, TJM, ILS, BDA, DICE, and CDDA). Similarly, for the Office + Caltech dataset with SURF features, it performs well compared to all other comparative methods except KUFDA and DICE.
- As KUFDA improves JGSA by considering the term Laplacian regularization, its average accuracy is much higher than other methods for the deep features of the Office + Caltech dataset, but less than the DICE method for most of the tasks in the PIE face dataset.
- For the PIE face and the Office + Caltech10 with SURF features datasets, DICE is performing better than all other methods (except STJML) because it is taking care of the intra-domain structure, especially for the target domain. However, its performance is abysmal for deep features of the Office + Caltech 10 dataset.
- Since our proposed method covers all the important objectives, as well as works on the projected subspaces of both the domains, the average accuracy of the proposed STJML method for all the tasks in all the considered datasets, is higher than all the other comparative methods. However, KUFDA beats our proposed algorithm for some tasks in the Office + Caltech dataset with deep features such as A→C, D→W, W→A, W→C, and W→C. Similarly, DICE beats the proposed method for eight tasks of the PIE face dataset.

### 4.8. Computational Complexity

Here, we analyze the computational complexity of Algorithm 1 by the O. The computational cost is detailed as follows: $O(D^{3}+nDd)$ for finding subspaces of both the source and the target domains, where D<n, i.e., Line 1; $O(n^{2}d)$ for constructing Laplacian matrix, i.e., Line 2; $O(n^{2}d)$ for computing kernel matrix, i.e., Line 3; $O(n^{2})$ for generating initial pseudo labels, i.e., Line 5; $O(t\left(dn^{2}+Cdn^{2}\right))$ for constructing marginal and conditional distribution matrices, i.e., Line 7; $O(tn^{2}d)$ for solving the generalized eigendecomposition problem with dense matrices, i.e., Line 9; $O(t\left(\sigma nn_{s}+\sigma nn_{t}\right))$ for computing $X_{s}^{\prime }$ and $X_{t}^{\prime }$ matrices, i.e., Line 11; $O(tn^{2})$ for generating pseudo labels, i.e., Line 12; $O(tn^{2})$ for computing the subgradient matrix, i.e., Line 13. In total, the computational complexity of Algorithm 1 is $O(D^{3}+nDd+n^{2}d+t\left(dn^{2}+Cdn^{2}+\sigma nn_{s}+\sigma nn_{t}+n^{2}\right))$. The complexity of this model can be greatly reduced by low-rank approximation.
**Algorithm 1:** Subspace based Transfer Joint Matching with Laplacian Regularization (STJML)
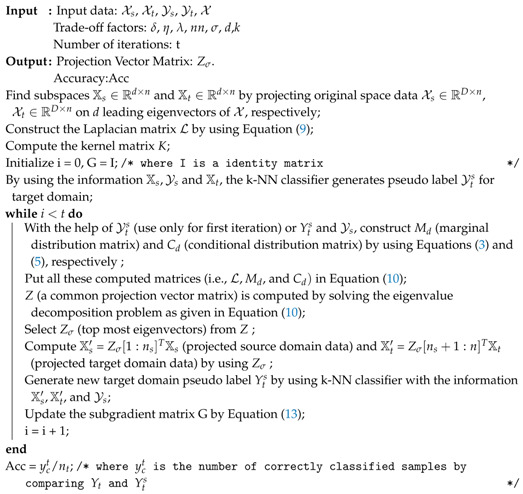


### 4.9. Running Time Analysis

To analyze the actual running time taken by each algorithm, we considered three tasks for the experiment such as C→A, C→D, and C→W, and reported the total time taken by the proposed and some other comparative methods to execute all the three tasks in Table 4. If we use the 1-NN classifier for the experiment, then the running time taken by it is minimal due to not performing any feature transformation. As TCA first presents feature transformation and then uses the 1-NN classifier for classification, it costs slightly more than the 1-NN classifier. Since GFK adds infinite subspaces for feature changes, it takes a longer time than the TCA method. JDA method improves the performance of TCA by considering the conditional distribution. To calculate the conditional distribution, we require a pseudo label for the target domain till the *t*th iteration. Therefore, its computational cost is higher than the TCA and GFK methods. The TJM method enhances JDA performance by considering additional instance-weighting terms. Thus, the computation cost of TJM also increases compared to JDA. Since the CORAL method first finds the co-variance matrices for both domains and then aligns the source domain to the target domain, its computation cost is higher than that of TJM, JDA, GFK, and TCA. JGSA includes two additional terms such as discriminative terms and subalignments and also uses two projection vector matrices for both domains. Therefore, JGSA consumes much time compared to other TJM, JDA, GFK, CORAL, and TCA. Since our proposed method improves the TJM by considering the subspace of both domains as well as adding the Laplacian term, it has a higher cost than TJM, but lower than JGSA.

## 5. Conclusions and Future Work

In this paper, we proposed a novel Subspace based Transfer Joint Matching with Laplacian Regularization (STJML) method for efficiently transferring knowledge from the source domain to the target domain. Because of jointly optimizing all the inevitable components, the proposed STJML method is robust for reducing the distribution differences between both domains. Extensive experiments on several cross-domain image datasets suggest that the STJML method performs much better than state-of-the-art primitive and transfer learning methods.

In the future, there are several ways through which we can extend our proposed method STJML, and some of them are:

Firstly, we will extend the STJML method to multi-task learning environments [42], where multiple tasks may contain some label samples. Thus, by using the label information of all tasks, all of them’ generalization performance can be enhanced.

Secondly, since the STJML method has many parameters and conducting manual parameter sensitive tests to find appropriate values is a hectic and time-consuming process. Furthermore, the STJML method uses the original features to find a common feature space. Still, the original features itself are distorted, then the STJML method will not become a robust classifier. Therefore, in the future, we will use the particle swarm optimization [43] method to select the appropriate value of each parameter and the proper subset of excellent features across both domains. So, the STJML method for selecting parameters will be strengthened, and its performance will also improve due to the elimination of distorted features.

Lastly, nowadays, there is increasing interest in neural-network-based learning models [44] due to their outstanding performance; we will also extend the STJML method to deep learning framework. In our deep learning STJML method, we will extract deep features concerning our proposed method overall objective function.

## Figures and Tables

**Table 1:** Accuracy (%) on the PIE face recognition dataset.

Tasks	Primitive Algorithms		Transfer Learning Algorithms																	
	NN	PCA	TCA [5]	GFK [29]	JDA [6]	CDML [32]	TJM [7]	TDA-AL [41]	CDDA [33]	BDA [35]	GA-DA [33]	DGA-DA [33]	JGSA [8]	RTML [22]	EMFS [37]	DICD [36]	LDADA [39]	KUFDA [16]	DICE [38]	STJML Proposed
PIE 1 5→7	26.09	24.80	40.76	26.15	58.81	53.22	29.52	35.97	60.22	23.98	57.40	65.32	68.07	60.12	61.8	73.0	34.5	67.67	84.1	82.32
PIE 2 5→9	26.59	25.18	41.79	27.27	54.23	53.12	33.76	32.97	58.70	24.00	60.54	62.81	67.52	55.21	58.8	72.0	44.9	70.34	77.9	73.22
PIE 3 5→27	30.67	29.26	59.63	31.15	84.50	80.12	59.20	35.24	83.48	48.93	84.05	83.54	82.87	85.19	86.8	92.2	61.5	86.06	95.9	94.65
PIE 4 5→29	16.67	16.30	29.35	17.59	49.75	48.23	26.96	28.43	54.17	24.00	52.21	56.07	46.50	52.98	52.6	66.9	35.4	49.02	66.5	62.99
PIE 5 7→5	24.49	24.22	41.81	25.24	57.62	52.39	39.40	38.90	62.33	49.00	57.89	63.69	25.21	58.13	59.2	69.9	31.4	72.62	81.2	81.27
PIE 6 7→9	46.63	45.53	51.47	47.37	62.93	54.23	37.74	49.39	64.64	24.00	61.58	61.27	54.77	63.92	64.5	65.9	34.9	74.34	74.0	82.1
PIE 7 7→27	54.07	53.35	64.73	54.25	75.82	68.36	49.80	53.26	79.90	48.97	82.34	82.37	58.96	76.16	77.9	85.3	53.5	87.86	88.6	91.91
PIE 8 7→29	26.53	25.43	33.70	27.08	39.89	37.34	17.09	36.95	44.00	24.00	41.42	46.63	35.41	40.38	44.3	48.7	26.4	61.70	68.8	69.54
PIE 9 9→5	21.37	20.95	34.69	21.82	50.96	43.54	37.39	34.03	58.46	49.00	54.14	56.72	22.81	53.12	53.8	69.4	38.2	73.91	78.8	77.16
PIE 10 9→7	41.01	40.45	47.70	43.16	57.95	54.87	35.29	49.54	59.73	23.95	60.77	61.26	44.19	58.67	59.8	65.4	30.5	72.56	76.7	80.9
PIE 11 9→27	46.53	46.14	56.23	46.41	68.45	62.76	44.03	48.99	77.20	48.97	77.23	77.83	56.86	69.81	70.6	83.4	60.6	86.96	85.2	90.68
PIE 12 9→29	26.23	25.31	33.15	26.78	39.95	38.21	17.03	39.34	47.24	24.00	43.50	44.24	41.36	42.13	41.9	61.4	40.7	69.85	70.8	71.01
PIE 13 27→5	32.95	31.96	55.64	34.24	80.58	75.12	59.51	42.20	83.10	49.00	79.83	81.84	72.14	81.12	82.7	93.1	61.3	90.00	93.3	95.55
PIE 14 27→7	62.68	60.96	67.83	62.92	82.63	80.53	60.58	63.90	82.26	23.96	84.71	85.27	88.27	8.92	85.6	90.1	56.7	88.40	95.00	93.01
PIE 15 27→9	73.22	72.18	75.86	73.35	87.25	83.72	64.88	61.64	86.64	24.00	89.17	90.95	86.09	89.51	88.2	89.0	67.8	84.62	92.3	90.37
PIE 16 27→29	37.19	35.11	40.26	37.38	54.66	52.78	25.06	46.32	58.33	24.00	53.62	53.80	74.32	56.26	57.2	75.6	50.4	75.24	81.1	82.65
PIE 17 29→5	18.49	18.85	26.98	20.35	46.46	27.34	32.86	32.92	48.02	49.00	52.73	57.44	17.52	29.11	49.4	62.9	31.3	54.05	73.8	63.17
PIE 18 29→7	24.19	23.39	29.90	24.62	42.05	30.82	22.89	37.26	45.61	23.89	47.64	53.84	41.06	33.28	45.1	57.0	24.1	67.46	71.2	75.5
PIE 19 29→9	28.31	27.21	29.90	28.49	53.31	36.34	22.24	36.64	52.02	24.00	51.66	55.27	49.20	39.85	55.9	65.9	35.4	70.77	74.1	76.83
PIE 20 29→27	31.24	30.34	33.64	31.33	57.01	40.61	30.72	38.96	55.99	48.94	58.82	61.82	34.75	47.13	59.6	74.8	48.2	76.78	81.8	82.9
Average	34.76	33.85	44.75	35.35	60.24	53.69	37.29	42.14	63.10	33.98	62.56	65.09	53.39	58.80	62.8	73.1	43.4	74.42	80.5	80.88

**Table 2:** Accuracy (%) on Office + Caltech dataset with VGG-FC6 features.

Tasks	Primitive Algorithms			Transfer Learning Algorithms												
	NN	PCA	SVM	TCA [5]	GFK [29]	JDA [6]	SA [15]	TJM [7]	CORAL [31]	CDDA [33]	ILS [34]	BDA [35]	JGSA [8]	KUFDA [16]	DICE [38]	STJML Proposed
A→C	70.1	76.49	74.2	80.14	77.73	82.01	77.1	82.45	79.0	82.1	78.9	80.23	81.12	85.12	83.6	84.14
A→D	52.3	59.87	51.7	65.60	59.23	70.06	64.9	72.61	67.1	68.2	72.5	64.97	68.78	78.34	66.0	78.34
A→W	69.9	69.15	63.1	76.94	73.89	83.72	76.0	82.71	74.8	78.1	82.4	76.61	78.30	80.16	76.6	91.11
C→A	81.9	86.43	86.7	86.63	86.01	88.10	83.9	85.80	89.4	86.5	87.6	86.01	86.22	89.83	89.5	90.51
C→D	55.6	61.14	61.5	69.42	62.42	72.61	66.2	75.79	67.6	66.1	73.0	66.88	77.07	80.13	69.9	87.89
C→W	65.9	74.23	74.8	74.91	74.91	80.67	76.0	77.96	77.6	77.1	84.4	75.93	76.61	87.83	79.8	89.15
D→A	57.0	67.43	58.7	75.15	68.58	77.13	69.0	80.79	75.6	82.6	79.2	74.32	86.95	85.21	83.2	86.84
D→C	48.0	58.50	55.5	69.18	59.57	70.52	62.3	74.44	64.7	76.1	66.5	69.72	78.09	80.38	78.7	82.63
D→W	86.7	95.59	91.8	96.61	95.93	97.62	90.5	96.94	94.6	93.7	94.2	97.63	97.62	98.87	95.8	98.3
W→A	62.4	75.15	69.8	80.27	79.01	84.2	76.6	82.25	81.2	86.5	85.9	80.79	90.81	91.56	88.8	90.51
W→C	57.5	69.01	64.7	75.24	70.16	74.79	70.7	78.45	75.2	80.1	77.0	76.22	76.66	84.12	82.0	83.17
W→D	83.9	94.90	89.4	93.63	94.90	96.81	90.4	94.90	92.6	92.8	87.4	92.36	92.99	100	88.1	100
Average	65.93	73.9	70.15	78.64	75.19	81.52	75.3	82.09	78.2	80.8	80.7	78.47	82.60	86.83	81.8	88.59

**Table 3:** Accuracy (%) on Office + Caltech dataset with SURF features.

Tasks	Primitive Algorithm			Transfer Learning Algorithms													
	1NN	PCA	SVM	GFK [29]	TCA [5]	JDA [6]	CORAL [31]	TJM [7]	SCA [30]	JGSA [8]	ARTL [14]	ILS [34]	RTML [22]	DICD [36]	LDADA [39]	DICE [38]	STJML Proposed
C→A	23.7	39.5	53.1	46.0	45.6	43.1	52.1	46.8	45.6	51.5	44.1	48.5	49.3	47.3	54.8	50.2	49.69
C→W	25.8	34.6	41.7	37.0	39.3	39.3	46.4	39.0	40.0	45.4	31.5	41.4	44.7	46.4	60.2	48.1	46.1
C→D	25.5	44.6	47.8	40.8	45.9	49.0	45.9	44.6	47.1	45.9	39.5	45.9	47.6	49.7	41.5	51.0	50.32
A→C	26.0	39.0	41.7	40.7	42.0	40.9	45.1	39.5	39.7	41.5	36.1	40.0	43.7	42.4	38.4	42.7	41.85
A→W	29.8	35.9	31.9	37.0	40.0	38.0	44.4	42.0	34.9	45.8	33.6	39.0	44.3	45.1	49.3	52.2	53.22
A→D	25.5	33.8	44.6	40.1	35.7	42.0	39.5	45.2	39.5	47.1	36.9	40.1	43.9	38.9	39.1	49.7	49.04
W→C	19.9	28.2	28.8	24.8	31.5	33.0	33.7	30.2	31.1	33.2	29.7	31.2	34.8	33.6	31.7	37.8	33.21
W→A	23.0	29.1	27.6	27.6	30.5	29.8	36.0	30.0	30.0	39.9	38.3	37.6	35.3	34.1	35.1	37.5	43.01
W→D	59.2	89.2	78.3	85.4	91.1	92.4	86.6	89.2	87.3	90.5	87.9	86.0	91.0	89.8	74.6	87.3	93.9
D→C	26.3	29.7	26.4	29.3	33.0	31.2	33.8	31.4	30.7	29.9	30.5	34.6	34.6	34.6	29.9	33.7	33.3
D→A	28.5	33.2	26.2	28.7	32.8	33.4	37.7	32.8	31.6	38.0	34.9	41.2	33.3	34.5	40.6	41.1	39.35
D→W	63.4	86.1	52.5	80.3	87.5	89.2	84.7	85.4	84.4	91.9	88.5	85.8	89.0	91.2	74.7	84.1	93.22
Average	31.4	43.6	41.1	43.1	46.2	46.8	48.8	46.3	45.2	50.0	44.3	47.6	49.3	49.0	47.5	51.3	52.18

**Table 4:** Running time complexity of proposed method (STJML) and some other methods.

Methods	Running Time (s)	Methods	Running Time (s)
1-NN	2.22	CORAL	47.05
TCA	3.42	JGSA	482.665
GFK	19.64	TJM	22.15
JDA	21.96	Proposed Method	72.74
